# Artificial Intelligence Predictor for Alzheimer’s Disease Trained on Blood Transcriptome: The Role of Oxidative Stress

**DOI:** 10.3390/ijms23095237

**Published:** 2022-05-07

**Authors:** Luigi Chiricosta, Simone D’Angiolini, Agnese Gugliandolo, Emanuela Mazzon

**Affiliations:** IRCCS Centro Neurolesi “Bonino-Pulejo”, Via Provinciale Palermo, Contrada Casazza, 98124 Messina, Italy; luigi.chiricosta@irccsme.it (L.C.); simone.dangiolini@irccsme.it (S.D.); agnese.gugliandolo@irccsme.it (A.G.)

**Keywords:** Alzheimer’s disease, data mining, machine learning, support vector machines, neural network, logistic regression, oxidative stress, transcriptomic analysis, microarray, blood

## Abstract

Alzheimer’s disease (AD) is an incurable neurodegenerative disease diagnosed by clinicians through healthcare records and neuroimaging techniques. These methods lack sensitivity and specificity, so new antemortem non-invasive strategies to diagnose AD are needed. Herein, we designed a machine learning predictor based on transcriptomic data obtained from the blood of AD patients and individuals without dementia (non-AD) through an 8 × 60 K microarray. The dataset was used to train different models with different hyperparameters. The support vector machines method allowed us to reach a Receiver Operating Characteristic score of 93% and an accuracy of 89%. High score levels were also achieved by the neural network and logistic regression methods. Furthermore, the Gene Ontology enrichment analysis of the features selected to train the model along with the genes differentially expressed between the non-AD and AD transcriptomic profiles shows the “mitochondrial translation” biological process to be the most interesting. In addition, inspection of the KEGG pathways suggests that the accumulation of β-amyloid triggers electron transport chain impairment, enhancement of reactive oxygen species and endoplasmic reticulum stress. Taken together, all these elements suggest that the oxidative stress induced by β-amyloid is a key feature trained by the model for the prediction of AD with high accuracy.

## 1. Introduction

Alzheimer’s disease (AD) is a neurological disease that impairs the normal life of millions of people worldwide and is considered a multifactorial complex pathology. It is defined on the basis of β-amyloid accumulation in the brain and neurofibrillary tangles. An early onset of the pathology is associated with a familial form of AD that can be explained by genomic alteration. The genes that, most often, are mutated in these AD cases are *APP* [1], *PSEN1* [2] and *PSEN2* [3]. Considering all AD cases, the familial form represents 4–6% of cases [4]. In most cases, however, a sporadic form of AD is detected with onset of pathology in older people (>65 years). Unfortunately, only neuroimaging techniques such as computed tomography (CT), magnetic resonance imaging (MRI) or positron emission tomography (PET) and clinical history are used to diagnose AD [5,6]. Moreover, it is also possible to evaluate AD biomarkers, specifically Aβ 1–42 and hyperphosphorylated tau, in cerebrospinal fluid (CSF) [7], but it requires an invasive procedure. Also, it is only possible to diagnose AD once the symptoms are quite evident, and no clinical procedure can reverse the status of the disease. For this reason, current research is focused on identifying biomarkers that can be detected, in a minimally invasive way, before the onset of symptoms. To assess the multifactoriality of the disease, transcriptomic studies aimed at finding differentially expressed genes (DEGs) between healthy subjects and patients affected by AD are frequently performed nowadays. Several studies confirm that alterations in the expression levels of genes related to AD are evident in the brain but can also be observable in the blood of patients [8]. The vast majority of annotated genes on the human reference genome are protein-coding genes, for which we have more information compared with noncoding RNAs (ncRNAs) [9]. In this sense, it is easy to understand the biological role of protein-coding genes, unlike ncRNAs. This imbalance of information in favor of coding RNA makes it possible to extrapolate more information about their biological role in the context of the disease being considered. Despite the huge amount of information available for protein-coding genes, the expression of ncRNAs is also altered in AD. Indeed, different studies show the importance of ncRNAs in the disease, and their involvement at the transcriptomic level is intensely studied [10]. Also, our research group has already identified several miRNAs in the AD brain compared with individuals without dementia [11]. In spite of these considerations, Li et al. show that coding and noncoding RNA are closely related to each other, and the study of both is important to have a sufficient overview in a transcriptomic analysis [12]. Nevertheless, even if several genes were identified as candidate biomarkers based on their levels of expression, there is no way to discriminate between a healthy subject and an individual with AD without clear expression of symptoms.

A method used to mine data from transcriptomics information, already applied with neurodegenerative diseases, is machine learning [13]. Machine learning methods are a subfield of artificial intelligence that can classify samples into different classes, minimizing the cost function of the trained model. Among them, supervised learning is a machine learning strategy in which the model is trained to learn labeled classes associated in clinics with healthy or sick conditions. Recently, inspecting the expression profile, this technique has been achieving excellent results in medical fields such as cancer [14,15]. The machine learning model can learn from different sources such as clinical and personal records, diagnostic images, biopsies or microarray data [16]. Some machine learning models are trained in neurodegenerative fields and also for AD. Most of them learned to predict AD through PET or other neuroimaging techniques [17]. Machine learning approaches have also been used to discriminate the disease at the epigenomic brain level [18]. Meanwhile, only a few of them are based on blood samples. The latter are usually trained on relatively small cohorts and are focused on ncRNAs such as miRNA [19,20,21]. The application of machine learning predictors to personalized medicine can help clinicians to make a diagnosis quickly with minimal errors. Using machine learning methods, it is also possible to discriminate which are the genes that are most responsible for the differences between the AD and healthy groups. The study of these genes makes it possible to discover and explore new possible biomarkers for pathology. Transcriptomic studies are not only focused on coding genes; also considering the implications of ncRNA in AD, many different research studies are based on miRNA, long non-coding RNA (lncRNA) or other classes of ncRNA. In light of the above, we designed a new machine learning predictor based on a microarray data platform that collects probes from 180 samples, 90 with AD and 90 without dementia (non-AD group), both for ncRNA and coding RNA. In particular, our model is able to predict AD with high accuracy starting from blood samples, discriminating genes that could be important markers for the early diagnosis of the pathology.

## 2. Results

### 2.1. Selected Features

**Figure 1:**
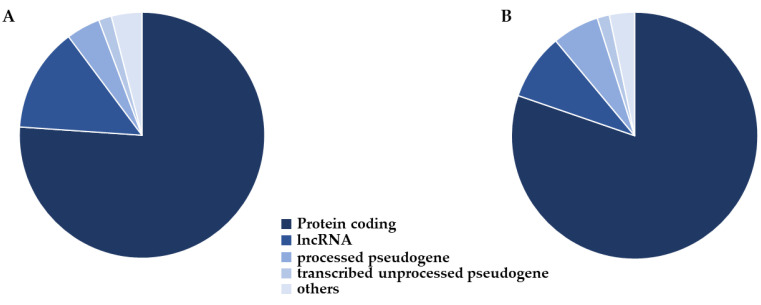
Biotype distribution in the dataset. The biotype characterization of the probes before (**A**) and after (**B**) feature selection. The two distributions are quite close to each other. Most of the probes are protein-coding. Also, large amounts of lncRNA, processed pseudogenes and transcribed unprocessed pseudogenes are present.

To look at the variance in the original datasets, we performed Principal Component Analysis (PCA). In particular, we searched for how many features were necessary to reach 95% of the variance, and we observed 117 components (Figure S1A). We observed that the “mutual_info_classif” score function gave better results, so we selected all the features whose score was higher than 3 variances after z-score normalization. We then recomputed the PCA using only the features selected, and 95% of the variance was reached with 87 components (Figure S1B). Since the amount of features was reduced, we computed the biotype distribution of the maintained ones. Thus, we plotted the biotype distribution on the selected features in Figure 1, and we observed a representation very similar to the original dataset.

Then, the feature normalization step was performed using RobustScaler and MinMaxScaler.

We enriched the selected features with Gene Ontology using the Panther website. We observed the over-representation of four ontologies in the category “biological process”, which are shown in Table 1. Particularly, “mitochondrial translation” and “mitochondrial gene expression” are very specific ontologies, while “cellular metabolic process” and “cellular process” are very general and not very informative.

Then, we also looked at the enriched features to see which of them was already associated with the “Alzheimer Disease” pathway in the KEGG database. Indeed, this database collects only manually curated information. The genes *ATF4*, *ATP5PF*, *AXIN1*, *CDK5*, *COX7A2L*, *EIF2AK2*, *ERN1*, *FZD2*, *HRAS*, *LRP6*, *NDUFB22*, *NDUFS5*, *NDUFS7*, *NFKB1*, *PSMA5*, *PSMD1*, *PSMD8*, *SDHA*, *SDHC*, *SDHD, SLC39A9*, *WNT4* and *XBP1* are the 23 selected features that are included in the pathway and highlighted in red in Figure S2.

### 2.2. DEGs

Furthermore, we computed the DEGs of the condition AD against non-AD. The differential analysis of the transcriptomic profiles revealed 4780 DEGs. Of these, 3562 are upregulated and more expressed in AD, whereas 1218 are downregulated and more expressed in non-AD. 

Thus, we enriched the 4521 DEGs for which the name of the probe was associated with a HGNC symbol. We observed that 167 ontologies are significative as “biological process”. Among them, we show in Table 2 the 10 ontologies with highest fold enrichment.

### 2.3. Differentially Expressed Features

**Figure 2:**
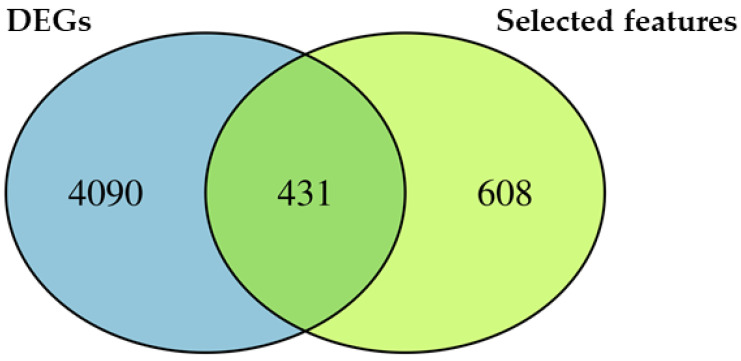
Venn diagram for distribution of DEGs and selected features. Among all the 4521 DEGs, the 4090 in the blue section are identified exclusively as DEGs. On the other hand, the 608 features in the green section are not identified as DEGs. Interestingly, 431 DEGs are highlighted in both the sections (intersection). Specifically, the genes included in the intersection are identified both as DEGs in the differential analysis and as important features by the model.

Additionally, we specifically looked at the genes identified both via feature selection and differentially expressed analysis. The Venn diagram in Figure 2 shows that 608 selected features are not identified as DEGs and 4090 DEGs are not inspected by the feature selection. Interestingly, the Venn diagram also highlights 431 genes that intersect the two categories. Indeed, they are identified as DEGs by the transcriptomic analysis but these genes are also used by the model as important features to make the prediction. In this sense, they are differentially expressed features.

Since the 431 differentially expressed features are identified both by differential expression and machine learning analysis, we speculate an important role for them as biomarkers. Thus, we enriched the 431 DEGs, and the first 10 ontologies with the highest fold enrichment are reported in Table 3.

**Figure 3:**
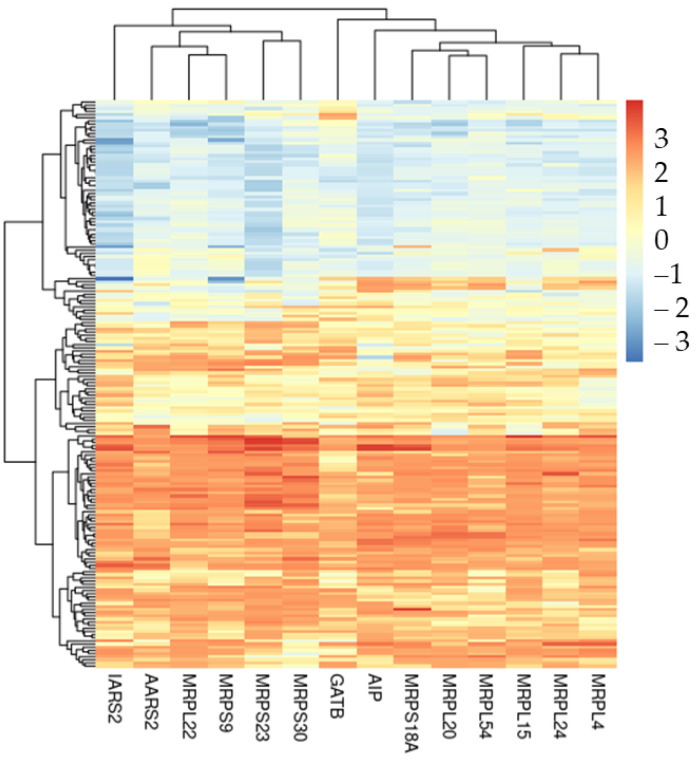
Heatmap of differentially expressed features in mitochondrial translation ontology. For each DEG inside this biological process, we plotted its expression level among the 180 individuals of the cohort.

Thus, we focus our attention on the first ontology based on fold enrichment, which is “mitochondrial translation”. The DEGs included in the ontology are represented in Table 4, and all of them are upregulated. Also, we have plotted in the heatmap in Figure 3 the expression level of these DEGs among all the samples in the cohort.

### 2.4. Training and Test Sets

**Figure 4:**
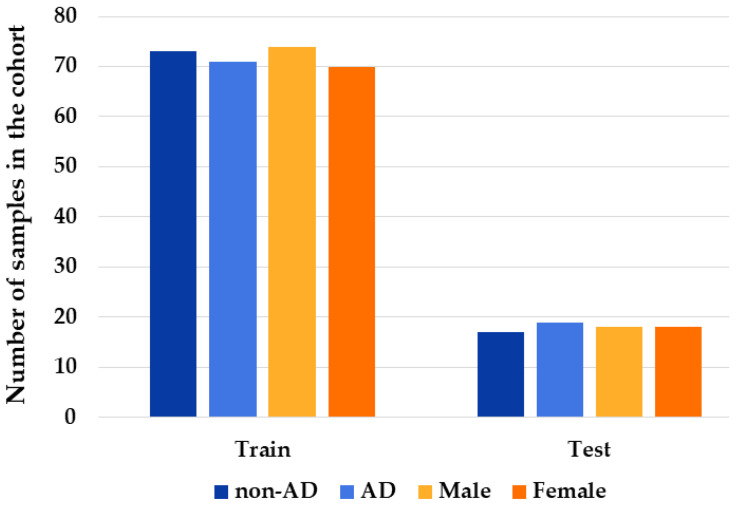
Dataset partition. The bar chart shows the distribution of statistics in training (on the left) and test (on the right) sets. The blue palette identifies the condition (non-AD or AD) of the different individuals. On the other hand, the orange palette shows the gender information (male or female). The chart highlights that the ratios of condition or gender are very similar in the training and test sets. The height of each bar shows the number of samples in the specific category.

The dataset with the selected features was used to build the training set and the test set. Specifically, we split the dataset into two parts in the proportion 80% (training)–20% (test). After the splitting, we computed the statistics of the training set to be sure that the proportion of personal characteristics was maintained. As shown in Figure 4, in the training set 73 are non-AD individuals and 71 were affected by AD, while 74 are male and 70 are female. On the other hand, the test set is composed of 17 non-AD individuals and 19 AD patients, among which 18 are males and 18 are females. Furthermore, the mean age of the training set is 76.65, whereas it is 75.67 in the test set. 

### 2.5. Model Evaluation

**Figure 5:**
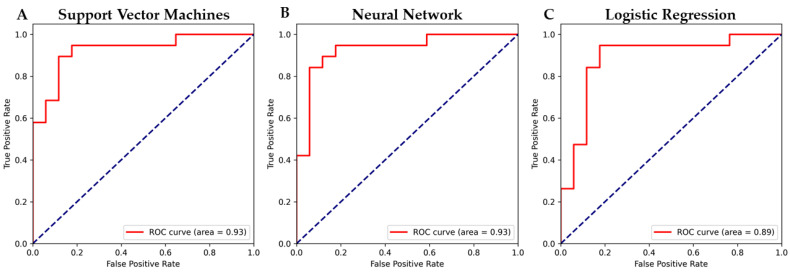
ROC–AUC for the models with higher scores. From left to right, the ROC–AUC is shown for support vector machines (**A**), neural network (**B**) and logistic regression (**C**). (**A**) shows that the ROC-AUC for the support vector machines model is 93%; (**B**) shows that the ROC-AUC for the neural network model is 93%; (**C**) shows that the ROC-AUC for the logistic regression model is 93%.

We trained eight different models using our dataset: logistic regression, linear discriminant analysis, decision tree classifier, Gaussian naive Bayes, k-neighbors classifier, random forest classifier, neural network and support vector machines. Additionally, for the model with the highest scores using default parameters, we performed a huge grid search inspection training the model on several different hyperparameters as shown in Table S1. Specifically, for the logistic regression method we tuned the penalty and consequently the L1 ratio during the elastic net. For the k-neighbors classifier, we tried different numbers of neighbors, and for the random forest classifier we used different estimators. The neural network was trained using different combinations of maximum iteration to converge, different hidden layers and different learning rates. The support vector machines model was validated on several different values of gamma and C for the three different kernel functions. At the end, the higher scores for level of accuracy and Receiver Operating Characteristic (ROC)-Area Under the Curve (AUC) were assigned to support vector machines (Figure 5A), logistic regression (Figure 5B) and neural network (Figure 5C).

In particular, the general highest scores shown in Table 5 were obtained for the support vector machines with C = 2^2^, gamma = 2^−7^ with the radial basis function kernel. High scores were also obtained by the logistic regression with L1 penalty and the neural network in 200 iterations, with 10 hidden layers at 0.001 learning rate. For each model we also computed the accuracy, F1, Matthews Correlation Coefficient (MCC), precision and recall.

## 3. Discussion

AD is a neurodegenerative disease that affects millions of people worldwide. To date, there is no way to confirm diagnosis of AD during life. The suggestion of AD diagnosis starts from clinicians when mild cognitive impairment flanks with problems in language, memory and other severe cognitive impairments. Clinicians can suppose AD after CT, PET, MRI or other imaging techniques. Each neuroimaging technique can focus on different aspects of the disease, but all of them lack specificity or sensitivity [22]. To date, brain autopsy is the only method that can be used to diagnose AD. For this reason, new predictive biomarkers are needed to reveal the early onset of AD antemortem. Levels of β-amyloid in the cerebrospinal fluid can be used as a biomarker for AD. Nevertheless, sampling the CSF is a highly invasive strategy [23]. Conversely, blood-based biomarkers are considered a very promising non-invasive strategy for early AD diagnosis [24]. Herein, we used a cohort of 90 AD patients and 90 individuals not affected by AD as control samples. Data for our cohort come from blood samples obtained in a non-invasive way. Specifically, the RNA was extracted from the blood, and the transcriptome was then sequenced through microarray. After a first step of data cleaning and manipulation, we split our cohort using 80% of the samples for the training set and 20% for the test set. Specifically, the mean ages of the samples in the training set and in the test set are quite close to each other. Additionally, as shown in Figure 4, the training set and test set are also similarly split in terms of disease condition and gender. This situation is required in order to build a reliable model that can equip healthcare staff in the clinical procedure. Thus, we trained the seven different machine learning models as shown in Table S1, testing whether any model was able to predict the two classes of AD patients and non-AD individuals. We observed that the linear discriminant analysis, decision tree classifier and Gaussian naive Bayes were not very good at discriminating between non-AD and AD. K-neighbors and random forest classifiers obtained better results but still not so impressive. The best performances were obtained by support vector machines, neural network and linear regression. Nevertheless, a huge grid search inspection was necessary. Indeed, with certain hyperparameters, even these three models performed very badly. We selected the best model on the basis of ROC score and accuracy. Then, we also computed F1, MCC, precision and recall scores. The ROC of the support vector machines reaches 93% (Figure 5A) and the accuracy reaches 88.8% with the radial basis function kernel. Also, the ROC of the neural network reached 93% (Figure 5B) but the accuracy was slightly lower (86.1%). Conversely, logistic regression has an accuracy of 89% but the ROC score is only 89% (Figure 5C). For this reason, the highest score is obtained by the support vector machines model. 

Since the scores of the predictor were quite high, we focused our attention on the kind of features selected to train the model. In particular, we investigated the specific biotype of the features that were used. We extracted this information for most of the probe using biomaRt. Figure 1A shows that most of the probe in the original dataset was made up of protein-coding genes. Then, a wide representation of lncRNA, processed pseudogenes and transcribed unprocessed pseudogenes is present. Thus, the feature selection procedure was trained using only miRNA, only lncRNA, only protein-coding or using all the features. We observed that the score of the protein-coding was quite close to the one that considers all the features. On the other hand, taking into consideration only the non-coding genes, the different scores drop a little bit. This consideration should also be taken into account because different models are trained just on the inspection of non-coding RNAs. Nevertheless, we have to take into consideration that the number of protein-coding genes overwhelms the number of non-coding RNAs in our set of features.

Once we had selected one specific gene biotype to run the study, or all the types, our models were not trained using the entire set of features. Indeed, we based our choice on PCA evaluation. Firstly, we searched for the number of linear combinations able to reach 95% of the variance. Now, we focus our attention on the models trained with all the biotypes since they reached the highest scores. In particular, these models count 899 protein-coding, 97 lncRNA and 69 processed pseudogenes along with other classes with less representation. Interestingly, the biotype distribution after feature selection, represented in Figure 1B, shows a very similar representation to the distribution of the features of the original dataset. Before feature selection, we had 117 components. We tried different “mutual_info_classif” and “f_classif” score functions to reduce the number of components. We observed that the final scores were quite close. Nevertheless, “mutual_info_classif” produced better results so we chose this function to evaluate the test set. After feature selection, we have 87 components. The high scores obtained by the model allow us to speculate that the selected features may be quite important to classify AD. For this reason, we want to inspect the biological meaning of our features. 

We used Gene Ontology enrichment to observe which processes, functions and components are related. Table 1 shows the very interesting results obtained by the enrichment of the selected features in the biological process ontology. Indeed, we observed two very specific ontologies related to the expression and translation of genes in mitochondria. In addition, we enriched the biological process of the 4521 DEGs obtained analyzing the transcriptomic profile of the whole cohort. Given the high number of DEGs, we found a high number of ontologies. Nevertheless, we found several ontologies related to mitochondrial and ribosome activity to be highly enriched (Table 2). To understand the biological meaning of the features used by the model, we also took advantage of the Venn diagram in Figure 2 to look at the features identified by the analysis of DEGs. Among the 431 differentially expressed features, Table 3 highlights the 10 most enriched ontologies, which are again related to the expression and translation of genes in mitochondria. Particularly, we focus our attention on the “mitochondrial translation” ontology, which is the most specific enriched ontology that we found. The ontology is represented by *MRPL54*, *MRPL22*, *MRPL24*, *MRPL15*, *MRPL20*, *MRPL4*, *MRPS18A*, *MRPS30*, *MRPS23*, *MRPS9*, *AARS2*, *IARS2*, *GATB* and *AIP*. Interestingly, all of them are upregulated. *MRPL54*, *MRPL22*, *MRPL24*, *MRPL15*, *MRPL20* and *MRPL4* are genes that encode for the large 39S mitochondrial ribosomal subunit. On the other hand, *MRPS18A*, *MRPS30*, *MRPS23* and *MRPS9* are components of the small 28S mitochondrial ribosomal subunit. Mitochondrial ribosomes, also known as mitoribosomes, are organelles active in the mitochondria that act in the matrix translating mitochondrial mRNA [25]. Gonçalves et al. showed that several mitochondrial ribosomal proteins (MRPs) are implicated in apoptosis and also associated with delayed cell proliferation and cell cycle progression. Also, MRPs have already been associated with neurodegenerative diseases such as Parkinson’s disease [26]. Sylvester et al. studied how MRPs can be implicated in mitochondrial disease [27]. Alterations in proteins involved in mitochondrial protein synthesis, including MRPs, were also described in mitochondria-associated membrane in the APP/PS1 mouse model of AD [28]. The inhibition of an MRP was found to reduce amyloid aggregation in models of AD, through an increase in mitochondrial proteostasis [29]. Interestingly, our study pointed out the importance of mitochondrial translation, highlighting that the genes involved, including MRPs, could also be important to discriminate AD samples from non-AD ones. These results indicate that they may represent new biomarkers and that their role in the pathology needs to be explored more deeply. Furthermore, *AARS2*, *IARS2* and *GATB* are involved in tRNA synthesis. In particular, *AARS2* belongs to the class-II aminoacyl-tRNA synthetase family and *IARS2* to the class-I aminoacyl-tRNA family. *GATB* is part of the glutamyl-tRNA(Gln) amidotransferase complex and biosynthesizes glutaminyl-tRNA(Gln). IAP encodes for the cytoplasmic Aryl Hydrocarbon Receptor Interacting Protein. Yano et al. showed that in cultured cells this protein acts in a multiprotein complex along with other mitochondrial proteins to enhance the import of mitochondrial preproteins [30].

Additionally, we also inspected the “Alzheimer Disease” pathway in the KEGG database. Specifically, we searched for whether any of the features we had selected was already known to be associated with AD. In particular, *PSMD1* and *PSMD8* encode for the 19S proteasomal subunit while *PDMA5* encodes for the 20S proteasomal subunit, part of the 26S proteasome. The aim of the 26S proteasome is the degradation of proteins marked by ubiquitins. Nevertheless, following oxidative stress, the proteasome’s activity is altered and the ability to degrade proteins can be lost [31]. In this way, the lack of degradation of proteins supports the accumulation of β-amyloid and hyperphosphorylated tau in AD. In turn, β-amyloid accumulation can aggravate the process. Indeed, β-amyloid peptides have been shown to inhibit the 26S proteasome’s proteolytic activities [32]. β-amyloid has the propensity to accumulate in the mitochondrial cristae through the translocase of the outer membrane (TOM) [33]. Sirk et al. showed that the accumulation of β-amyloid in the TOM hinders the crossing of proteins encoded by the nucleus inside the mitochondria [34]. As positive feedback, the aforementioned mitochondrial ribosome proteins, which are encoded from nuclear DNA, cannot cross into the mitochondria. In turn, the mitochondrial DNA cannot encode the proteins of the mitochondrial complexes, impairing the electron transport chain and enhancing the production of free radicals and, consequently, oxidative stress [35]. Among the features used to train the model, we found *NDUFB11*, *NDUFS5*, *NDUFS7*, *SDHA*, *SDHC*, *SDHD*, *COX7A2L* and *ATP5PF*, which encode for mitochondrial complexes I, II, IV and V. Mitochondrial complexes physiologically produce reactive oxygen and nitrogen species, which in AD cannot be fully erased, leading to oxidative stress [36]. *NDUFB11*, *NDUFS5* and *NDUFS7* encode for complex I and *COX7A2L* for complex IV. These complexes are strongly associated with the oxidative stress condition that seems linked to β-amyloid accumulation [37]. Additionally, the accumulation of β-amyloid inside the mitochondria seems, in AD, to alter the stability of complex V, whose subunit is encoded by *ATP5PF*, inducing reactive oxygen species (ROS) production. Differently to the other complexes, complex II is encoded only from nuclear genes. Here, *SDHA*, *SDHC* and *SDHD* features are used. Interestingly, it has already been associated with neurodegenerative disease, and it seems to produce ROS through reverse electron transfer [36]. Furthermore, in AD, ROS also lead to stress of the endoplasmic reticulum (ER) [38,39] with the activation of the unfolded protein response (UPR). Indeed, to reduce the stress, IRE1α (encoded by *ERN1*), XBP1 and ATF4 are recruited. Interestingly, these proteins were used to train our model. Specifically, IRE1α is a stress sensor protein involved in the canonical UPR pathway that activates XBP1. XBP1 is a transcriptional factor that upregulates several genes involved in protein and organelle quality control. On the other hand, the transcriptional factor ATF4 is encoded to induce an antioxidant response [40].

A strong connection is present among all these elements because the mitochondrial ribosome plays a key role in the translation of mitochondrial mRNA responsible for proteins involved in mitochondrial complexes. We speculate that impairment of mitochondrial ribosomes causes alterations in mitochondrial translation, leading to an abnormal expression of mitochondrial DNA and, as a consequence, to dysfunctions of the mitochondrial complexes. This leads to an oxidative stress condition because mitochondria are responsible for 90% of the endogenous ROS. In association with oxidative stress, the accumulation of β-amyloid and unfolded proteins, caused by proteasome inhibition, leads to ER stress and UPR. Thus, dysfunctional mitochondria could represent a major source of oxidative imbalance present in AD and also in the early stage of the pathology [41,42,43].

The results we have obtained in this work open up for us the possibility of future studies to improve the model and facilitate the early diagnosis of AD. First of all, the model could be designed to include additional personal information for the samples in the cohort. In this way, genomics information, clinical record and imaging could be used to increase the accuracy of the model. Secondly, blood samples for each patient could be used additionally to train the model to learn how the transcriptomic profile changes over time. 

## 4. Materials and Methods

### 4.1. Microarray Dataset Selection

We searched the AD dataset from blood in the Gene Expression Omnibus (GEO) repository [44]. We then filtered for Homo sapiens (9606), microarray expression profile on the relative filter section. We selected the experiment with BioProject ID PRJNA338435. The original dataset is composed of 180 individuals, among whom 90 are AD patients and 90 are individuals without AD. The mean age of the AD patients is 77.68, while 75.23 is the mean age of the other individuals. Also, the cohort is composed of 92 males and 88 females. No information about other comorbidities was provided. 

### 4.2. Sample Preparation

As reported by the authors who deposited the data, the total RNA of the people was extracted from the peripheral blood cells. Then, the high quality of the RNA was preserved by following the manufacturer’s instructions for the Ribopure Blood RNA Purification kit (Ambion Technologies, Austin, TX, USA). After treatment with an on-column agent for DNase digestion agent, through the NanoDrop-1000 spectrophotometer the RNA was quantified, and the Agilent 2100 Bioanalyzer (Agilent Technologies, Santa Clara, CA, USA) was used to monitor it. Then, following the instructions of the manufacturer, RNA was labeled with Cyanine-3 (Cy3) using the One-Color Low Input Quick Amp Labeling kit (Agilent). After purification with the RNeasy column (QIAGEN, Valencia, CA, USA), dye was incorporated and the NanoDrop ND-1000 spectrophotometer was used to perform the last quality check. The sample hybridization was performed on Agilent Whole Human Genome Oligo Microarrays (G2534A). After washing, the Agilent microarray scanner G2505B (Agilent Technology) provided the scans using 8 × 60 k array slides as settings for one-color scan.

### 4.3. Matrix Reconstruction and DEG Analysis

The original dataset sequenced using the microarray technology provided a matrix with the samples for columns and the probes for rows. In detail, the matrix had 180 columns, among which 90 were the patients with AD and 90 were the individuals without AD. On the rows, 42,545 were the probes, and they are related both to coding and non-coding genes. Firstly, we proceed with a normalization step of the features on R (version 4.1.2) using the package limma (version 3.50.1) [45] of Bioconductor [46]. Limma uses different statistical principles in a way that is adapted for large-scale expression studies. It provides the functions “backgroundCorrect” and “normalizeBetweenArrays”. The function “backgroundCorrect” allows correction of the expression of the intensities in the microarray. On the other hand, “normalizeBetweenArrays” normalizes their expression, and we used the quantile normalization method, which is the most efficient strategy [47]. Then, the type of molecules for each probe was obtained using the package biomaRt [48], which provided a link to the Ensembl database [49] for the GRCh38.p13 version of the human genome. The details of the gene biotype were obtained using the attribute “gene_biotype”. Additionally, with biomaRt the information about the name symbol of the probes was retrieved through the attribute “hgnc_symbol”. Among all the features, some have the same symbol associated with different biotypes. We kept just one of them. Also, we searched for all the probes that biomaRt associates with the same gene. Additionally, we excluded probes that are not associated with biomaRt. Finally, we observed that among the 33,416 features, 21,488 are unique genes and for 2228, “hgnc_symbol” does not result assigned to any symbol. Along these lines, we collected all the probes with non-unique gene symbols, and we replaced them with their mean value for samples. The final number of probes is 23,716, among which most are protein-coding as shown in Figure 1A. Several biotypes are also lncRNA, processed pseudogenes or transcribed unprocessed pseudogenes.

Thus, the final matrix, stored in Table S2, has 23,721 columns and 183 rows. There are 180 samples (on the rows), and there are 23,716 probes (on the columns). The first three rows are the header, the gene symbol and the biotype information. The first fifth columns are the row names, geo accession, age, gender and disease condition.

Then, we used limma to observe how the transcriptomic profiles of AD patients differ from those of the non-AD ones. We did not use any fold change cutoff, but we did filter out all the genes with *p*-value corrected by Benjamini–Hochberg higher than 0.05.

### 4.4. Feature Selection and Normalization

Firstly, we split the original dataset into two parts where the larger one (80% of the samples) was used as the training set and the smaller one (20% of the samples) as the test set. To reduce the number of features and train the model, we used Python 3.8. In particular, the scikit-learn library (version 1.0.2) was used to perform the Principal Component Analysis decomposition. It is based on the numpy (version 1.20.3) [50] and scipy (version 1.8.0) [51] libraries. PCA was used to visualize the amount of variability inside our original dataset. Thus, we searched for the number of components that represent 95% of the variance of our dataset. To reduce the number of components, reducing the possibility of overfitting, we adopted the feature selection procedure. Specifically, we used univariate selection strategies to avoid the redundancy of the features. Due to the nature of our dataset, we used both “mutual_info_classif” and “f_classif” as score functions. Then, we chose the number of features to keep for building the model. We normalized the score of the features obtained by the univariate selection through z-score normalization. After normalization, we used only the feature whose normalized score was higher than 3 variances over the mean (z-score > 1.96). Finally, we normalized the features to be used to train the models using the package preprocessing of scikit-learn. Firstly, we normalized using the RobustScaler function, which removes the median values according to the quantile range. Then, we used the MinMaxScaler function to shrink all the values in a range from 0 to 1. 

### 4.5. Feature and DEG Enrichment

We then enriched the features selected and the DEGs with Gene Ontology using the Panther [52] website. We used the default parameters. Thus, Fisher’s exact test was used to compute the statistical relevance, while we took advantage of the false discovery rate to correct the *p*-value. Additionally, we used the package “KEGGREST” on R and “pathview” of Bioconductor to observe which of the selected features are already known to be associated with the “Alzheimer Disease” pathway (hsa05010) on the KEGG [53] database.

### 4.6. Model Construction and Hyperparameter Tuning

We then built different models based on 8 different classifiers: logistic regression, linear discriminant analysis, decision tree classifier, Gaussian naive Bayes, k-neighbors classifier, random forest classifier, neural network and support vector machines. Also, we built the models using several different combinations of the hyperparameters (Table S1).

For each model, we collected the following parameters: accuracy, recall, precision, F1-score, Matthew’s correlation coefficient and the ROC. We provided each parameter both for training and test sets to understand whether the model was first of all able to learn from the data and to avoid overfitting.

## 5. Conclusions

Machine learning models can be a very useful instrument to support clinicians in the diagnosis of neurodegenerative diseases such as AD. Herein, we demonstrate that a support vector machines model trained on transcriptome data extracted from blood samples can reach an ROC score of 93% and accuracy of 88%. Interestingly, the predictor uses features involved in oxidative stress. Specifically, the mitochondrial translation process mediated by mitochondrial ribosomes is suggested to have a pivotal role in discriminating AD from non-AD samples. Furthermore, β-amyloid accumulation could impair the electron transport chain, increase oxidative stress and induce ER stress. In this way, oxidative stress seems to be a key feature that characterizes the model for the prediction of the early onset of AD with high accuracy.

## Figures and Tables

**Table 1:** Gene ontologies enriched for the selected features.

Gene Ontology ID	Gene Ontology Description	Genes	Fold Enrichment	False Discovery Rate
GO:0032543	mitochondrial translation	19	3.86	2.02 × 10^−2^
GO:0140053	mitochondrial gene expression	21	3.31	3.46 × 10^−2^
GO:0044237	cellular metabolic process	392	1.20	4.65 × 10^−2^
GO:0009987	cellular process	777	1.12	2.65 × 10^−6^

All of the enriched gene ontologies in the “biological process” category with statistical significance are shown. The ontologies are sorted by fold enrichment.

**Table 2:** Gene ontologies enriched for the DEGs.

Gene Ontology ID	Gene Ontology Description	Genes	Fold Enrichment	False Discovery Rate
GO:0070129	regulation of mitochondrial translation	17	3.39	2.39 × 10^−2^
GO:0062125	regulation of mitochondrial gene expression	18	3.10	4.78 × 10^−2^
GO:0000154	rRNA modification	21	2.83	3.08 × 10^−2^
GO:0000387	spliceosomal snRNP assembly	21	2.76	3.75 × 10^−2^
GO:0140053	mitochondrial gene expression	76	2.73	1.58 × 10^−8^
GO:0032543	mitochondrial translation	59	2.73	1.39 × 10^−6^
GO:0006476	protein deacetylation	30	2.46	2.03 × 10^−2^
GO:0016575	histone deacetylation	28	2.41	3.78 × 10^−2^
GO:0001510	RNA methylation	43	2.33	1.93 × 10^−3^
GO:0042273	ribosomal large subunit biogenesis	33	2.29	2.10 × 10^−2^

The 10 enriched gene ontologies in the “biological process” category with statistical significance and higher fold enrichment are shown. The ontologies are sorted by fold enrichment.

**Table 3:** Gene ontologies enriched for the DEGs included as selected features.

Gene Ontology ID	Gene Ontology Description	Genes	Fold Enrichment	False Discovery Rate
GO:0032543	mitochondrial translation	14	7.20	6.25 × 10^−5^
GO:0140053	mitochondrial gene expression	16	5.97	1.36 × 10^−4^
GO:0008380	RNA splicing	23	3.32	9.90 × 10^−4^
GO:0006412	translation	23	3.16	1.78 × 10^−3^
GO:0043043	peptide biosynthetic process	24	3.08	1.83 × 10^−3^
GO:0006397	mRNA processing	26	3.06	8.87 × 10^−4^
GO:0016071	mRNA metabolic process	35	3.04	6.37 × 10^−5^
GO:0006518	peptide metabolic process	28	2.70	2.65 × 10^−3^
GO:0006396	RNA processing	39	2.36	1.38 × 10^−3^
GO:0043603	cellular amide metabolic process	35	2.26	7.80 × 10^−3^

The 10 enriched gene ontologies in the “biological process” category with statistical significance and higher fold enrichment are shown. The ontologies are sorted by fold enrichment.

**Table 4:** DEGs included in the “mitochondrial translation” biological process.

Gene	Non-AD Mean Expression	AD Mean Expression	Fold Change	q-Value
*MRPL54*	0.56	1.14	0.58	1.82 × 10^−2^
*MRPS23*	0.29	1.35	1.06	2.01 × 10^−3^
*MRPL22*	0.45	1.33	0.88	1.99 × 10^−3^
*MRPS18A*	0.58	1.28	0.70	1.20 × 10^−2^
*MRPL24*	0.40	1.16	0.75	4.19 × 10^−3^
*MRPS9*	0.33	1.19	0.86	4.52 × 10^−3^
*MRPS30*	0.38	1.44	1.05	3.68 × 10^−4^
*AARS2*	0.33	1.21	0.88	1.27 × 10^−3^
*IARS2*	0.34	1.02	0.68	4.30 × 10^−2^
*AIP*	0.50	1.17	0.66	2.65 × 10^−2^
*GATB*	0.48	1.20	0.72	2.02 × 10^−3^
*MRPL15*	0.49	1.23	0.74	7.25 × 10^−3^
*MRPL20*	0.57	1.13	0.56	3.85 × 10^−2^
*MRPL4*	0.34	1.25	0.91	1.28 × 10^−3^

The fold change for the analysis was computed using limma. All the values are rounded to the second decimal digit. The q-Value stands for the *p*-Value after post-hoc Benjamini-Hochberg correction.

**Table 5:** Models that obtain the best performance.

Model	ROC	Accuracy	F1	MCC	Precision	Recall
Support Vector Machines	0.93	0.89	0.90	0.78	0.86	0.95
Neural Network	0.93	0.86	0.87	0.72	0.85	0.90
Logistic Regression	0.89	0.89	0.90	0.78	0.86	0.95

Among all the models, the support vector machines, neural network and logistic regression methods achieved the best performance after grid search.

## Data Availability

The data presented in this study are openly available in the NCBI Sequence Read Archive at BioProject accession number PRJNA338435.

## Supplementary Materials

The following supporting information can be downloaded at: https://www.mdpi.com/article/10.3390/ijms23095237/s1.
