# Supplementary material for: Artificial Intelligence Predictor for Alzheimer’s Disease Trained on Blood Transcriptome: The Role of Oxidative Stress
Source: Int J Mol Sci. 2022 May 7;23(9):5237. doi: 10.3390/ijms23095237 (PMC9104709; doi:10.3390/ijms23095237)
Supplement: Supplementary file 1 [file ijms-23-05237-s001.zip › Table S1.pdf]

**Table S1.** List of models used during training set procedure

| Model | Parameter 1 | Parameter 2 | Parameter 3 |
|---|---|---|---|
| Logistic regression | solver: liblinear, saga | penalty: l1, l2, elasticnet | l1_ratio=0.1, 0.3, 0.5, 0.7, 0.9 |
| Linear discriminant analysis | - | - | - |
| Decision Tree Classifier | - | - | - |
| Gaussian Naive Bayes | - | - | - |
| K-Neighbors Classifier | n_neighbors: 2, 5, 10 | - | - |
| Random Forest Classifier | n_estimators: 10, 100, 1000 | - | - |
| Neural Network | max_iter: 100, 200, 500 | hidden_layer_sizes: 2, 10, 100 | learning_rate_init=0.0001, 0.001, 0.01, 0.1 |
| Support Vector Machines | kernel: rbf, sigmoid, ploy | C: $2^{-5}$, $2^{-4}$, $2^{-3}$, $2^{-2}$, $2^{-1}$, $2^{0}$, $2^{1}$, $2^{2}$, $2^{3}$, $2^{4}$, $2^{5}$, $2^{6}$, $2^{7}$, $2^{8}$, $2^{9}$, $2^{10}$, $2^{11}$, $2^{12}$, $2^{13}$, $2^{14}$, $2^{15}$ | gamma: $2^{-15}$, $2^{-14}$, $2^{-13}$, $2^{-12}$, $2^{-11}$, $2^{-10}$, $2^{-9}$, $2^{-8}$, $2^{-7}$, $2^{-6}$, $2^{-5}$, $2^{-4}$, $2^{-3}$, $2^{-2}$, $2^{-1}$, $2^{0}$, $2^{1}$, $2^{2}$, $2^{3}$ |

We specified the functions and the combination of parameters and hyperparameters used during the grid search to train the different models. The hyphen is put if the parameter is not used. The parameters not specified are used as set on default.
