# Supplementary figures and images for: Artificial Intelligence Predictor for Alzheimer’s Disease Trained on Blood Transcriptome: The Role of Oxidative Stress
Source: Int J Mol Sci. 2022 May 7;23(9):5237. doi: 10.3390/ijms23095237 (PMC9104709; doi:10.3390/ijms23095237)

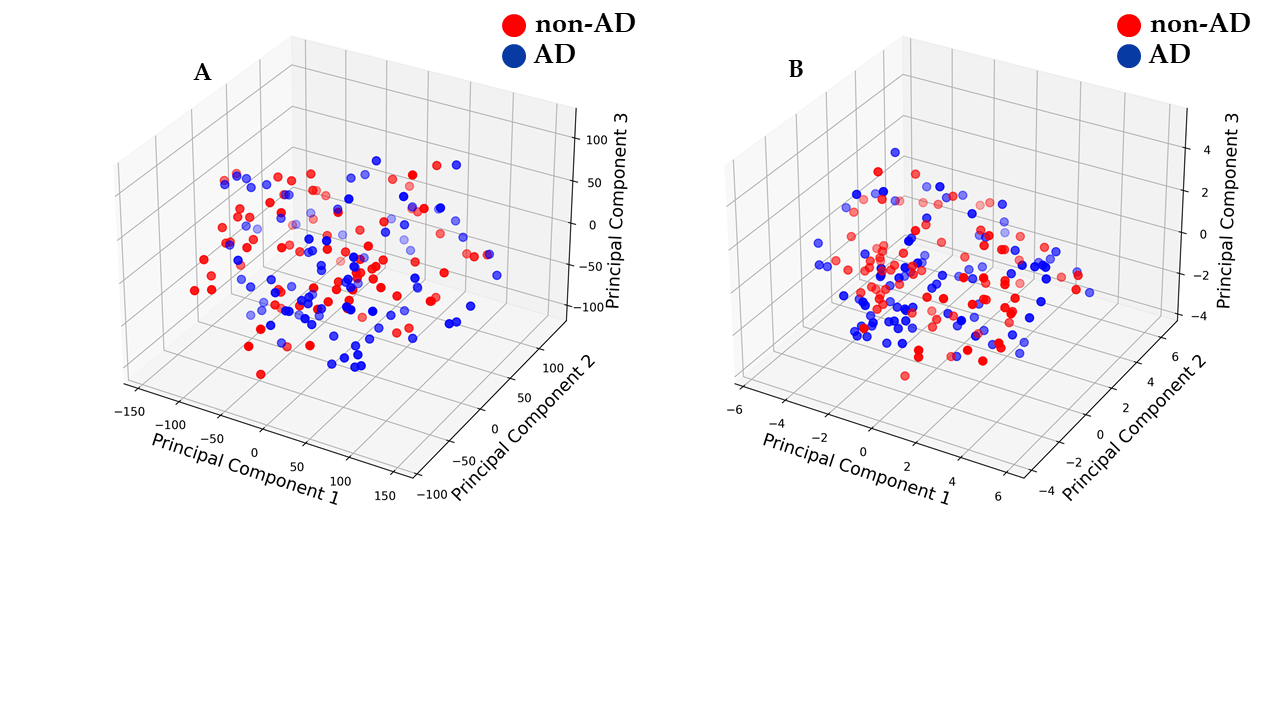

Supplement: Supplementary file 1 [file ijms-23-05237-s001.zip › Figure S1.tif]

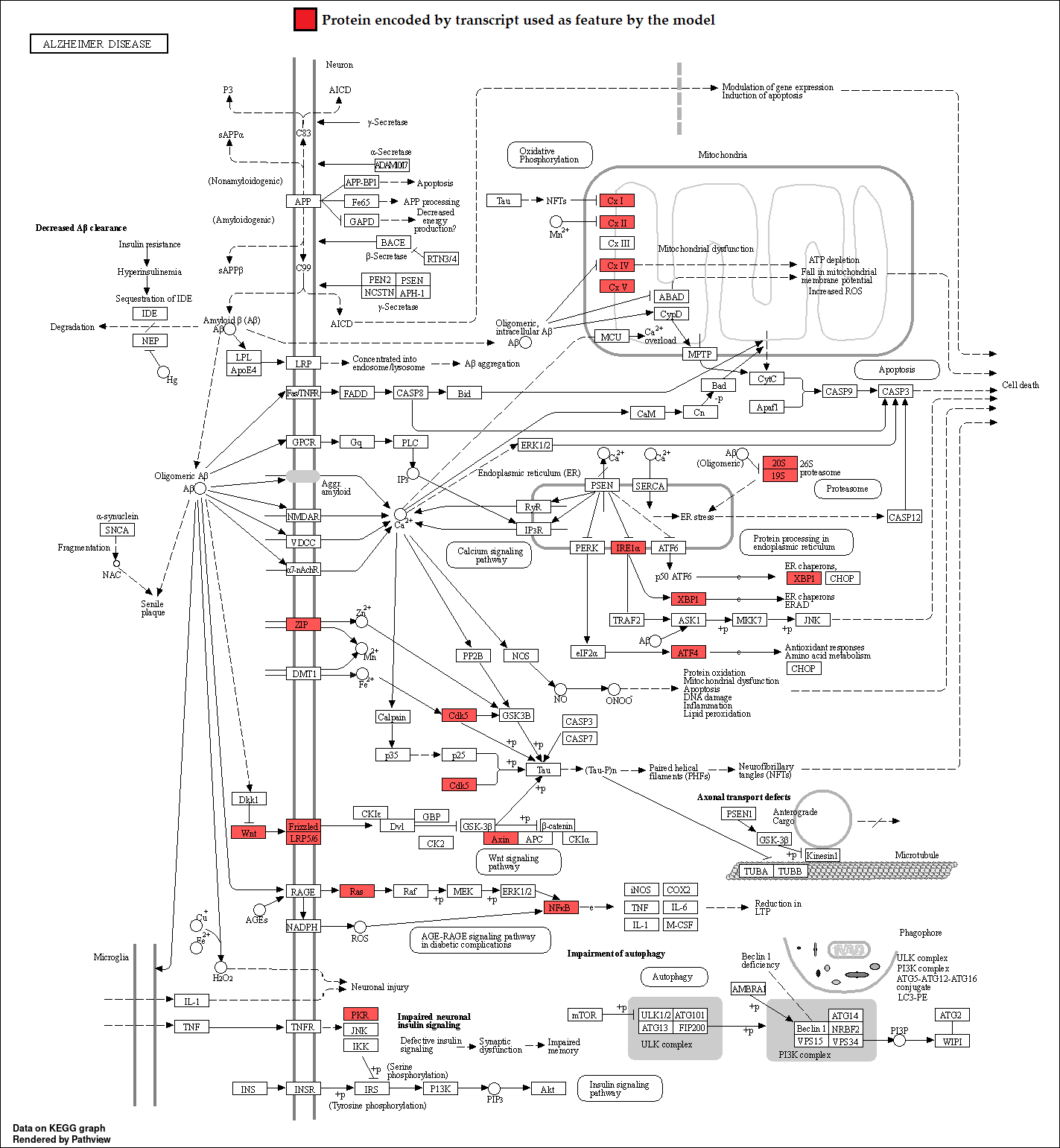

Supplement: Supplementary file 1 [file ijms-23-05237-s001.zip › Figure S2.tif]
